# The Relationship Between Depression, Burnout, and Suicide Among Healthcare Professionals: A Scoping Review

**DOI:** 10.1111/wvn.70037

**Published:** 2025-05-13

**Authors:** Nam Nguyen, Elizabeth Spooner, Pamela O'Balle, Hannan Ashraf, Karen Heskett, Sidney Zisook, Judy E. Davidson

**Affiliations:** ^1^ Department of Psychiatry University of California San Diego USA; ^2^ School of Medicine University of California San Diego USA; ^3^ San Diego School of Medicine, Department of Psychiatry Distinguished Professor, University of California San Diego USA

**Keywords:** burnout, compassion fatigue, depression, emotional exhaustion, healthcare workers, mental health, occupational stress, suicidal behaviors, suicide prevention, workplace well‐being

## Abstract

**Background:**

Burnout and mental health concerns are prevalent among healthcare workers. Female physicians, nurses, and healthcare staff are at a higher risk of suicide than the general population. Burnout and depression have been known to coexist with suicidal ideation and behaviors.

**Aims:**

To identify what is known about the relationship between burnout and depression in the context of suicide among healthcare workers.

**Methods:**

Registered scoping review of English language articles indexed to CINAHL, PubMed, and PsychInfo databases with date of publication prior to March 5, 2024.

**Results:**

The review yielded nine eligible studies, all employing observational or descriptive methodologies. Depression was found to be a predictor of suicidal ideation. While burnout was associated with depressive symptoms and found to coexist with suicidal ideation, it was not predictive of ideation. Emotional exhaustion and depersonalization were key components of burnout linked to depression. No studies were found exploring survivorship factors in healthcare professionals. Suggested prevention strategies that need to be tested include mindfulness and cognitive‐behavioral skills training, improved workplace conditions, addressing loneliness, and fostering resilience.

**Linking Evidence to Action:**

Interventional studies are needed to test strategies addressing burnout, depression, suicidal behaviors, and survivorship of suicide attempts. Depression should be considered and evaluated when healthcare workers exhibit symptoms of burnout. Moreover, the Socio‐economic Model of Suicide Prevention (i.e., SESM) can be used to categorize suicide prevention measures in healthcare. Burnout and depression interact to influence mental health outcomes among healthcare professionals, with depression playing a more significant role in predicting suicidal ideation. Despite the demonstrated relationships, critical gaps in knowledge exist in understanding survivorship and in the development and testing of effective interventions. Future interventional multisite research is needed using validated tools to identify best practices in suicide prevention for healthcare professionals.

## 1 Background

The Surgeon General, American Hospital Association, Academy of Nursing, and National Academy of Medicine all agree that suicide prevention strategies are needed within healthcare organizations to reduce suicides of healthcare professionals (American Hospital Association 2023; Office of the Surgeon General 2022; Schimmels et al. 2023; Wakefield et al. 2021). The risk of suicide has been established, especially among females in the healthcare professions, female nurses, and healthcare support staff (Davidson et al. 2024; Olfson et al. 2023).

Previous reports have suggested that a relationship may exist between burnout, depression, and suicidal thoughts and behaviors (STB) such as ideation, suicide attempts, suicide, or self‐harm. In one retrospective study of healthcare professionals seeking mental health support, a third of those with burnout or depression experienced both burnout and depression. Either burnout alone or depression alone was a predictor of psychological distress and suicidal ideation compared to when neither of these existed, with depression being a stronger predictor (Zisook et al. 2022). While burnout and depression are two different constructs, both have been reported to coexist with suicidal ideation or behaviors (Verkuilen et al. 2021; Zisook et al. 2022). Because of this association, leaders and scientists have been cautioned that there may be a risk in assessing burnout alone without also considering depression (Verkuilen et al. 2021; Zisook et al. 2022). While previous research has separately examined the prevalence of burnout, depression, or suicide, the relationship between these constructs has not been comprehensively explored (Ryan et al. 2023).

**FIGURE 1:**
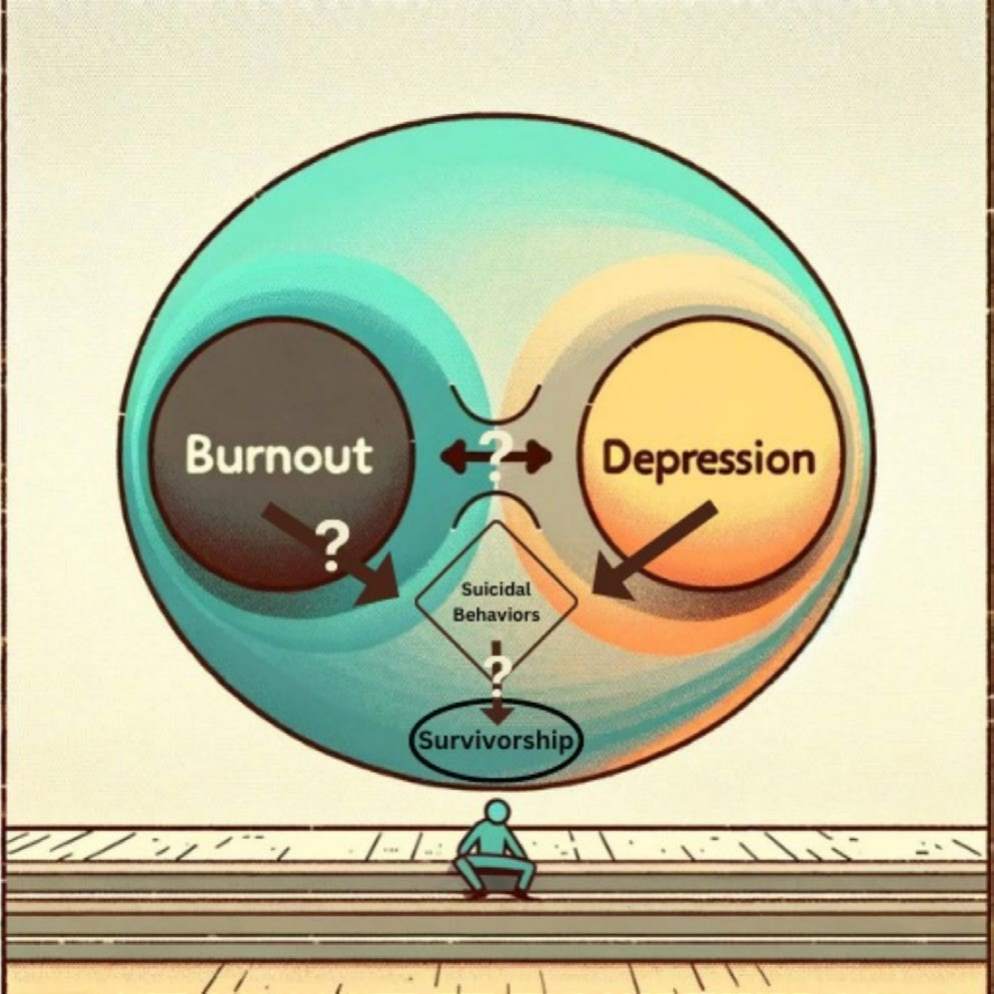
Scoping review model. It was the assumption of this research team that the relationship between depression and suicide is well documented, where the relationship between burnout and depression in the context of suicide has not been summarized. Similarly, factors associated with survivorship have not been summarized.

Before embarking on further research in this niche area of practice, it is important to determine what is known and not known about the relationships between depression and burnout in the context of STB. Furthermore, because the relationship between burnout and depression is not completely understood, the best methods of suicide prevention for those with both conditions do not exist. Previously, researchers have focused on suicide among healthcare professionals by looking back and examining incidence or death narratives following a suicide (Davidson et al. 2024; Davis et al. 2021; Olfson et al. 2023). At this juncture, it would be prudent to move toward studying survivorship, factors associated with surviving STB, which can be used toward building prevention models. A scoping review maps the existing literature, identifies areas lacking in comprehensive research, and suggests directions for future studies (Peters et al. 2020; Sutton et al. 2019). This scoping review is aimed at exploring what is known about the relationship between burnout, depression, and STB among healthcare professionals (Figure 1). The ultimate goal of focusing on the relationships between burnout, depression, STB, and suicide is to cultivate the knowledge needed to inform best practices in suicide prevention.

### 1.1 Research Questions

The research questions were developed according to the Kao method (Population, Context, Concept) (Kao et al. 2017). Population was defined as *healthcare professionals*, context as *burnout and depression*, and concept as *suicide*.

#### 1.1.1 Primary Research Question

What is the relationship between [burnout], [depression], and [STB] among healthcare professionals?

#### 1.1.2 Secondary Research Questions

What are the existing suicide prevention strategies tested within healthcare settings for professionals experiencing burnout and depression?

What is known about the survivorship of healthcare professionals who experience burnout and depression prior to suicidal behavior?

### 1.2 Variables

#### 1.2.1 Dependent Variable(s)

The primary dependent variable was (1) suicidal behavior, including suicidal ideation, self‐harm, suicide attempts, and suicide.

The secondary dependent variables were (2) survivorship of suicidal behavior and (3) best practices in suicide prevention.

#### 1.2.2 Independent Variable(s)

The two independent variables were (1) burnout and (2) depression.

### 1.3 Expectations

It is standard with scoping reviews to declare a priori expectations similar to hypotheses (Peters et al. 2020; Sutton et al. 2019). We anticipated that little would be known about the relationship between burnout and depression within the context of STB among healthcare professionals or about the survivorship of healthcare professionals who experience burnout and depression. We anticipated that burnout or depression would be discussed in the context of suicide but would not be consistently addressed simultaneously. We expected there would be differences between what is known about subgroups within this broad population; there may be a difference between physicians, nurses, advanced practice providers, pharmacists, and healthcare support staff. We anticipated the Socio‐ecological Model of Suicide Prevention (SESM), first developed by the Centers for Disease Control and Prevention (CDC 2023) and later refined by Cramer and Kapusta (2017), could be used to map results and would be appropriate for use in the healthcare professional population.

## 2 Methods

### 2.1 Ethics

This study was approved by the Institutional Review Board (#810644).

### 2.2 Eligibility

**FIGURE 2:**
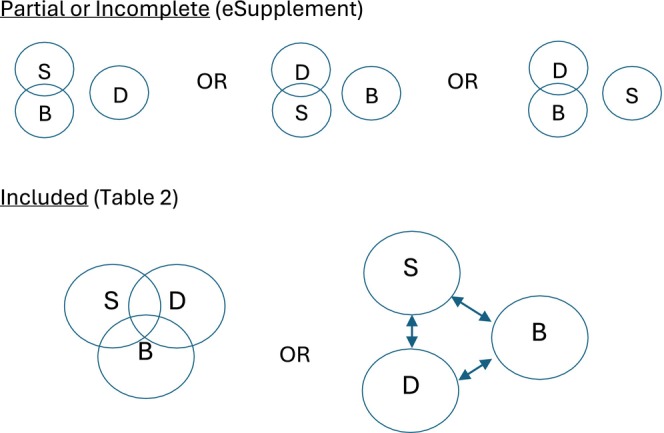
Inclusion criteria visual model. Partial or Incomplete (eSupplement). Included (Table 2). Suicide, suicidal thoughts, behaviors, self‐harm, survivorship, suicide; B, burnout; D, depression. Ven diagrams overlap, association; Arrows, predictions.

The inclusion criteria for this scoping review encompassed studies focused on healthcare professionals (physicians, nurses, pharmacists, and healthcare support staff) that examined the relationship between burnout, depression, and STB. While suicidal ideation versus suicide attempts may be treated differently clinically, little is known about these constructs among healthcare professionals. We cast a broad net to evaluate what is known about both within the definition of STB (Klonsky et al. 2021). Further, self‐harm is a known risk for suicide, yet a different construct than suicidal ideation or attempt. As others have done before, for the purposes of this study we elected to keep self‐harm within the definition of STB (Groves et al. 2024a, 2024b; Large et al. 2021). Eligible studies were required to be original research or performance improvement reports, available as full‐text English‐language articles, and available in selected databases prior to March 5, 2024. Studies that focused exclusively on burnout or depression without addressing suicidal behaviors, as well as editorials, opinion pieces, narrative reviews, and those involving populations outside of healthcare professionals were excluded. Studies that addressed the relationship between burnout and suicide without mentioning the relationship among depression, suicide, and burnout were also excluded. Studies that looked at the three variables separately in the context of a dependent variable or intervention other than suicide were excluded. Articles that mentioned all three variables but examined the relationship between only two of the three concepts of burnout, depression, and suicidal behaviors were considered partial or incomplete, sorted separately, and are presented in the Supporting Information. Due to the complexity of this analysis, a visual model is provided (Figure 2).

### 2.3 Search Strategy

A comprehensive search was conducted in three major electronic databases: PubMed (PubMed.gov), CINAHL (EBSCO), and PsycINFO (ProQuest). Each database was accessed through its respective interface. The search strategy was developed with the assistance of a health sciences librarian, ensuring the appropriate use of Boolean operators, MeSH terms, and key search terms for each database (Table 1 and S1). Gray literature and review papers were also considered by scanning the reference lists of relevant review papers to capture potentially missed citations, but were excluded from the final extraction phase. Duplicates were removed in Covidence.

**TABLE 1:** Search strategy for the relationship between depression, burnout, and suicidal behaviors among healthcare professionals.

Concept	MeSH headings	Keywords
Burnout	Burnout, professional [MeSH]	Burnout, burnout syndrome, professional burnout
Depression	Depression [MeSH]	Depressive disorder, clinical depression, major depression, mental health [MeSH], Depress^a^
Suicidal behaviors	Suicidal ideation [MeSH], suicide, attempted [MeSH], self‐injurious behavior [MeSH]	Suicide attempt^a^, self‐harm, suicide ideation^a^, suicidal ideation^a^
Healthcare workers	Health personnel [MeSH], nurses, physicians, pharmacists [MeSH]	Healthcare professionals, physicians, nurses, pharmacists, medical staff

Abbreviation: MeSH, Medical subject headings.

^a^ Truncation symbol that will retrieve any similar word variations.

### 2.4 Article Selection

The title and abstract of each article were independently reviewed by two investigators applying the inclusion and exclusion criteria. The full research team was divided into two smaller teams, each consisting of 2 members. One member from each team was paired to screen a subset of the articles. After independent screening, the reviewers discussed discrepancies with their teammates and reached a consensus. Each team's decision was presented to the full research team prior to acceptance. Final decisions regarding the inclusion or exclusion of studies were documented in Covidence (Covidence 2024).

Following the title and abstract screening, the same process was applied to the full‐text review. Each team reviewed half of the selected full‐text articles, and findings were discussed iteratively to synthesize what was known versus identify gaps in the literature. Two additional screening stages for overlap in variables were conducted using Excel. In the third screening stage, articles were categorized into exclude, partial/incomplete, or keep. The project mentor, independent from the research team, was available to resolve any discrepancies during the screening process, ensuring consistency and accuracy in the study selection.

### 2.5 Data Extraction

Data extraction was conducted using a Microsoft Excel document to systematically collect and organize information from the studies included and stored in a password protected Microsoft Teams file. Extracted data included key study characteristics: study design, level of evidence, population/sample characteristics, country of origin, variables of interest, instruments used, sample size, key findings (including statistical results), and limitations. The Johns Hopkins Evidence‐Based Practice Professional Model and Guidelines were used during extraction (Dang et al. 2022). Additionally, studies included in the final analysis were also evaluated for proposed interventions according to the SESM public health framework for suicide prevention: societal, community, relational, and individual (Cramer and Kapusta 2017). Interventions recommended at the level of the profession were mapped to the societal level, and institutional or organizational‐level interventions were mapped to the community level.

Two teams, each consisting of two reviewers, were responsible for extracting data from the selected articles. In each team, both reviewers independently read the assigned articles and discussed their findings with each other to determine whether the article should be extracted. If discrepancies arose, they were discussed within the entire team to reach a consensus. In cases where consensus could not be achieved, a third reviewer or mentor was consulted to resolve the issue. This review adhered to Kao et al.'s (2017) method of scoping reviews, and the report was written according to the Preferred Reporting Items for Systematic Reviews and Meta‐Analyses (PRISMA) guidelines extension for scoping reviews to enhance transparency and methodological rigor (Tricco et al. 2018). The methods for this scoping review were registered in the Open Science Framework (OSF) on March 5, 2024 (Nguyen et al. 2024).

## 3 Results

### 3.1 Search Results

**FIGURE 3:**
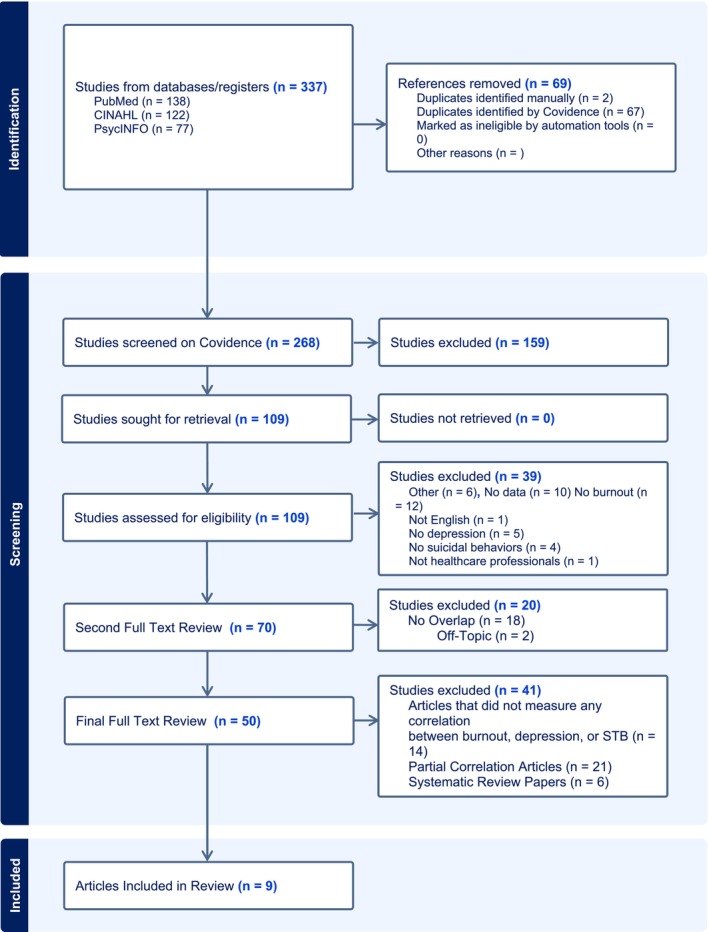
PRISMA diagram. Systematic review papers were excluded from the final review as the topics did not fit the inclusion criteria for this scoping review. Additionally, reference lists did not yield new relevant articles.

The search yielded a total of 337 records. After removing 69 duplicates, 268 records remained for screening. Based on title and abstract review, 159 records were excluded, leaving 109 full‐text articles for assessment. Of these, 39 articles were excluded, resulting in 70 records. Upon a second full‐text review, an additional 20 articles were excluded, leaving 50 articles. In the final full‐text review, nine published studies met the inclusion criteria. Most of the articles excluded were opinion pieces, commentaries, narrative reviews, or did not address the intersection of burnout, depression, and suicidal behaviors. A PRISMA diagram of this process is provided in Figure 3.

### 3.2 Characteristics of Studies

Our search identified nine eligible studies that examined burnout, depression, and suicide among healthcare professionals (Table 2). These studies used various observational and descriptive methods to assess mental health outcomes across different populations, including physicians, nurses, and allied health professionals. No studies of experimental design were found. Commonly used tools for measurement included surveys and standardized mental health assessment instruments (Table 2).

**TABLE 2:** Evidence summary.

Author	Title	Population and measurement tools	Relationship between burnout, depression, and suicidality	Limitations/weaknesses
Samuelsson et al. (1997)	Suicidal feelings and work environment in psychiatric nursing personnel	Population: nurses in Sweden Sample size: 197 (81% response rate) Measurements: 1. Burnout: Swedish abridged version of the Maslach burnout inventory (MBI) 2. Depression: the hopelessness scale (Beck et al. 1974) 3. Suicidality: assessed via five questions by Paykel (1974): “have you felt that life is not worth living, wish you were dead, and thoughts of suicide, seriously considered suicide, and have you attempted suicide.”	Examined the relationship between environmental burnout/depression and suicidality; a significant negative correlation of −0.395 (*p* < 0.0001) was observed. Various subscales of suicidality were measured in relation to burnout and depression, showing the following correlations: death wish (−0.508), thoughts of suicide (−0.598), feeling life is not worth living (−0.220), considering suicide (−0.630), and attempted suicide (−0.087). A negative work environment is correlated with increased burnout/depression which in turn increases the risk for suicidality.	Did not assess for depression directly instead assessed for hopelessness. Measured burnout and depression on the same scale.
Menon et al. (2020)	Association of physician burnout with suicidal ideation and medical errors	Population: physicians in the United Sates Sample size: 1354 (11.4% response rate) Measurements: 1. Burnout: Stanford professional fulfillment index (PFI), the Maslach burnout inventory–human services survey for medical personnel (MBI), and the Mini‐Z burnout survey. 2. Depression: patient‐reported outcomes measurement information system (PROMIS) depression 4‐item short form. 3. Suicidality: response to the question, “during the past 12 months, have you had thoughts of taking your own life?”	Prior to adjusting for depression, there was an 85% increase in the likelihood of suicidal ideation (SI) for each standard deviation (SD) increase in the burnout scale (odds ratio [OR], 1.85; 95% CI, 1.47–2.31). After adjusting for depression, burnout was no longer linked to increased odds for SI (OR, 0.85; 95% CI, 0.63–1.17). Findings were consistent across all burnout subscales (PFI interpersonal disengagement, PFI work exhaustion, MBI depersonalization, and MBI emotional exhaustion). Every SD increase in depression was associated with 202% increase in SI odds (OR, 3.02; 95% CI, 2.30–3.95).	Low response rate, unable to conduct separate subgroup analyses of attending physicians and residents.
Williford et al. (2018)	Multiple‐institution comparison of resident and faculty perceptions of burnout and depression during surgical training	Population: physicians in the United States Sample size: 147 (59.8% response rate) Measurements: 1. Burnout: Maslach burnout inventory (MBI) 2. Depression: patient health questionnaire‐9 depression screen (PHQ‐9). 3. Suicidality: response to item 9 of the PHQ‐9 > or = 1.	Burnout was associated with a 6‐point increase in the PHQ‐9 depression scale (coefficient [SE], 6.08 [1.41]; *p* < 0.001). 28 of the 58 residents experiencing burnout also met criteria for depression (*p* = 0.03). No significant association was found between burnout and suicidal ideation. Among the participants, 9 residents experienced both suicidal ideation and burnout, 49 had burnout without suicidal ideation, and none reported suicidal ideation alone. 18 participants reported neither suicidal ideation nor burnout. Although all residents who reported suicidal ideation also met criteria for both burnout and depression, no significant association was found between the level of training and burnout, depression, or suicidal ideation.	Limited demographic. Did not examine the perceived causative factors of burnout among residents.
Yun et al. (2023)	Associations among the workplace violence, burnout, depressive symptoms, suicidality, and turnover intention in training physicians: A network analysis of nationwide survey	Population: physicians in republic of Korea Sample size: 1981 (14.6% response rate) Measurements: 1. Burnout: Maslach burnout inventory (MBI). 2. Depression: patient health questionnaire‐9 (PHQ‐9) 3. Suicidality: response to item 9 of the PHQ‐9 > or = 1.	Burnout was not associated with suicidality. Depression was associated with suicidal ideation. Depressive symptoms were associated with the intention to quit their job. Workplace violence (lateral and patient) was associated with burnout, depression, and intention to quit their job.	The figures were published pixilated and difficult to read and interpret. The explanations of the findings were unclear.
Bai et al. 2022	The prevalence and risk factors for major depression and suicidal ideation in medical residents based on a large multicenter cross‐sectional study using the propensity score‐matched method	Population: physicians in China Sample size: 1343 (86.48% response rate) Measurements: 1. Burnout: Maslach burnout inventory‐general survey (MBI‐GS). 2. Depression: patient health questionnaire‐9 (PHQ‐9) 3. Suicidality: response to item 9 of the PHQ‐9 > or = 1.	Burnout subscale with high depersonalization and poor personal accomplishment increased the odds of major depression (OR = 1.068, OR = 0.963) and suicidal ideation (SI) (OR = 1.097, OR = 0.962). Risk factors for major depression and SI were found to be poor sleep quality, lower optimism of psychological capital, as well as increased burnout.	Cross‐sectional research unable to show causation between variables
Kleinhendler‐Lustig et al. (2023)	Burnout, depression, and suicidal ideation among physicians before and during COVID‐19 and the contribution of perfectionism to physicians' suicidal risk	Population: physicians in Israel Sample size: 246 (response rate not noted) Measurements: 1. Burnout: Maslach burnout inventory (MBI). 2. Depression: patient health questionnaire‐8 (PHQ‐8) 3. Suicidality: suicide behaviors questionnaire‐revised (SBQ‐R)	Identified a positive relationship between current suicidal ideation and factors such as depression, burnout, and maladaptive perfectionism both prior to and during the COVID‐19 pandemic. Showed burnout had a significant positive effect on depression, with no substantial differences between the two time periods (B = 0.002, SE = 0.04, *p* = 0.966). The influence of burnout on suicidal ideation was not significant, and time did not moderate this effect (B = 0.04, SE = 0.04, *p* = 0.390). The study suggests implementation of intervention programs to address maladaptive perfectionism to lessen the risk of suicidal ideation.	Small sample size. Unable to calculate response rate. Data were collected early in the COVID‐19 pandemic, which may have affected results. No subgroup analysis among distinct types of physicians.
Ofei‐Dodoo et al. (2019)	Burnout and quality of life among active member physicians of the medical society of Sedgwick County	Population: physicians in the United States Sample size: 197 (44.6% response rate) Measurements: 1. Burnout: abbreviated Maslach burnout Inventory (MBI‐9). 2. Depression: Primary Care Evaluation of Mental Disorders (PRIME MD) Patient Health Questionnaire (PHQ‐2) 3. Suicidality: response to the question, “During the past 12 months, have you had thoughts of taking your own life?”	The presence of burnout (100% vs. 46.9%, *p* < 0.01) increased the likelihood to have suicidal ideation by 2.13 times and depression (72.6% vs. 30.4%; *p* < 0.001) by 2.38 times. All 9 of the physicians who reported SI also screened positive for burnout. Physicians with burnout, depression, fatigue, and suicidal thoughts had a strong desire to leave the medical field.	Short‐form screening tools were used instead of longer or diagnostic measures limiting the depth of variable characterization. Did not control for depression when assessing the impact of burnout.
Lebares et al. (2018)	Burnout and stress among US surgery residents: Psychological distress and resilience	Population: Physicians in the United States Sample Size: 566 (response rate not noted) Measurements: 1. Burnout: Abbreviated Maslach Burnout Inventory (MBI) 9‐items. 2. Depression: Modified version of the Patient Health Questionnaire‐9 (PHQ‐9). 3. Suicidality: Measured by asking, “In the last two weeks, how often have you been bothered by thoughts that you would be better off dead or harming yourself in some way?”	Found strong association between burnout, (specifically emotional exhaustion), depersonalization, and increased risks of depression and suicidal ideation. Physicians with high emotional exhaustion were 5.78 times more likely to experience suicidal ideation (*p* < 0.0001) and 4.82 times more likely to report moderate to severe depression (*p* < 0.0001). Depersonalization was linked to a 2.18‐fold increase in suicidal ideation (*p* = 0.0165) and a 2.36‐fold increase in depression (*p* = 0.0009). The findings show elevated levels of both emotional exhaustion and depersonalization are positively correlated with depressive symptoms and suicidal ideation further highlighting the severe mental health impacts of burnout in the medical profession.	Unable to determine accurate response rate.
Zisook et al. (2022)	Relationship between burnout and major depressive disorder in health professionals: A HEAR report	Population: mixed healthcare workers in the United States Sample size: 2281 (7.6% response rate) Measurements: 1. Burnout: abbreviated Maslach burnout inventory (MBI) 3‐items. 2. Depression: patient health questionnaire‐9 (PHQ‐9). 3. Suicidality: measured with questions about suicidal ideation, planning, and self‐harm, with a score ≥ 1 indicating the presence of such thoughts or behaviors. Participants also rated intense affective states associated with suicide risk, with a score of ≥ 2 indicating the presence of these emotional states.	Participants with burnout and depression reported significantly higher rates of suicidal ideation and behaviors (18.9% had suicidal thoughts, 6.9% had suicidal plans, 2.7% had suicidal behaviors, and 10.2% had a history of suicide attempts) (OR of 11.53 for suicidal thoughts, 9.40 for suicidal plans, 8.22 for self‐harm behaviors, and 1.83 for suicide attempts.) Participants with depression alone showed lower odds ratios for suicidality: 0.79 for suicidal thoughts, 0.63 for suicidal plans, 0.48 for self‐harm behaviors, and 0.53 for suicide attempts. Burnout alone had no increase in suicidality but did increase intense affective states.	Low response rate. The single‐item measure for burnout, “feeling burned out from your work,” is not psychometrically validated, and the modified PHQ‐9 used for depression screening is not a diagnostic tool for major depressive disorder (MDD).

In these studies of healthcare professionals, a variety of assessment tools were used to measure the correlation between burnout, depression, and suicidal attempts, thoughts of self‐harm, or ideation. All studies were classified as third‐level nonexperimental research on the Hopkins evidence scale. Some common tools included the Maslach Burnout Inventory‐Human Services Survey, which assessed emotional exhaustion, depersonalization, and personal accomplishment (Maslach and Jackson 1981). The Stanford Professional Fulfillment Index, Mini‐Z Burnout Survey, and the PROMIS Depression Short Form were also frequently used to measure burnout (Cella et al. 2010, 2019; Linzer et al. 2016; Schalet et al. 2016; Trockel et al. 2018). Depression and suicidal ideation were assessed using the PHQ‐9, with suicidal risk measured by the Suicide Behaviors Questionnaire‐Revised (SBQ‐R) (Kroenke et al. 2001; Osman et al. 2001). Additional tools, such as the PHQ‐2 and PHQ‐8, were employed to screen for depression, alongside measures like the Frost Multidimensional Perfectionism Scale (Kroenke et al. 2009; Whooley et al. 1997).

### 3.3 Primary Research Question: Relationship Between Burnout, Depression, and Suicide

Though we intended to evaluate studies including actual self‐harm or completed suicide, none were found. All studies that met inclusion criteria focused on suicidal ideation, thoughts of self‐harm, suicide attempt, burnout, and depression. Across multiple studies focusing on the relationship between burnout, depression, and suicidal behaviors among healthcare workers, burnout and depression were strongly associated with suicidal ideation. Depression emerged as a more predictive indicator of suicidal ideation than burnout (Bai et al. 2022; Kleinhendler‐Lustig et al. 2023; Lebares et al. 2018; Ofei‐Dodoo et al. 2019; Samuelsson et al. 1997; Zisook et al. 2022). For example, in one study, burnout was significantly correlated with suicidality (−0.395), including subscales such as thoughts of suicide (−0.598) and death wishes (−0.508), highlighting the strong connection between these mental health factors and suicidality (Samuelsson et al. 1997). However, after adjusting for depression, burnout's direct effect on suicidal ideation was often reduced or eliminated, implying depression plays a mediating role in the relationship between burnout and suicidality (Menon et al. 2020; Yun et al. 2023).

Many studies identified that burnout alone was more closely linked to depression than suicidal ideation. For instance, burnout was associated with a 6‐point increase on the PHQ‐9 depression scale, but there was a weak association between burnout and suicidal ideation (Menon et al. 2020; Williford et al. 2018). Moreover, components of burnout, such as emotional exhaustion and depersonalization, are particularly critical in their association with depression. Physicians experiencing high emotional exhaustion were 5.78 times more likely to report suicidal ideation and 4.82 times more likely to screen positive for moderate to severe depression, further emphasizing the emotional toll of burnout (Bai et al. 2022; Lebares et al. 2018; Menon et al. 2020; Yun et al. 2023).

Depression was consistently found to be a strong predictor of suicidal ideation. Depressive symptoms such as sadness, feelings of worthlessness, and psychomotor changes were significantly associated with suicidal ideation, while burnout did not show such a direct connection (Kleinhendler‐Lustig et al. 2023; Yun et al. 2023). Conversely, individuals that experienced both burnout and depression tended to have higher odds of participating in both suicidal thoughts and behaviors compared to those that only experienced burnout or depression, indicating that the combination of these two factors may increase the risk of suicidal behaviors (Ofei‐Dodoo et al. 2019; Zisook et al. 2022).

### 3.4 Secondary Research Questions: Interventions and Survivorship

No studies examined interventions or survivorship directly. However, characteristics were identified that might shape future prevention efforts. Dispositional mindfulness, defined as the innate ability to pay attention to one's thoughts, emotions, and experiences in a nonreactive way, has been shown to have a buffering effect against perceived stress and burnout among healthcare workers (Lebares et al. 2018). Individuals with higher dispositional mindfulness tended to have a decreased risk of burnout, stress, and distress symptoms (Lebares et al. 2018). Moreover, the risk‐reducing benefits associated with higher dispositional mindfulness were equivalent to, or better than, those found with higher trait resilience (Lebares et al. 2018). This is particularly important as mindfulness can be dispositional or inherent, meaning it can be taught to health professionals. Consequently, the findings suggest mindfulness training may be an effective tool for stress resilience and prevention in high‐stress, high‐performance groups. Moreover, to reduce suicidal ideation in medical residents, a balanced approach to psychological capital development that fosters hope, self‐efficacy, and resilience should be included in prevention and treatment strategies (Bai et al. 2022).

Lateral violence was associated with burnout, depression, and intention to leave, but not with suicidal ideation (Yun et al. 2023). Specifically, the results indicate peer‐on‐peer verbal or physical abuse strongly contributed to increased levels of emotional exhaustion and depressive symptoms, which correlate with a higher likelihood of trainees considering leaving their positions. However, no association was observed between lateral violence and suicidal ideation. Additionally, while survivorship factors were listed a priori as secondary outcomes, no studies were found that directly reported these factors (Yun et al. 2023).

The proposed implications from each study were mapped to the SESM model. Specific interventions include wellness training, psychotherapy, workplace and training method modifications, and targeted efforts to address burnout (Table 3). Additionally, the studies emphasize the importance of providing links to community resources, enhancing societal support during crises, and recognizing the broader societal impact of burnout.

**TABLE 3:** Implications mapped to the socio‐economic model of suicide prevention.

Author	Individual implications	Community implication	Relational implications	Societal implications
Samuelsson et al. (1997)	Not discussed	Mental health, burnout, suicidal behavior among psychiatric nursing personnel influenced by work environment	Not Discussed	Not Discussed
Menon et al. (2020)	Chronic stress leads to psychological distress, emphasizing need for resilience and support strategies	Institutional role in burnout among healthcare workers	Not Discussed	Not Discussed
Bai et al. (2022)	Risk factors for depression in residents: poor sleep, low optimism, high exhaustion	High burnout levels in medical community; need for support	Not Discussed	Not Discussed
Williford et al. (2018)	Burnout and depression among residents; barriers to care	Support for surgical community through community‐level interventions	Impact on healthcare team dynamics	Public health concern regarding burnout's effect on healthcare quality
Yun et al. (2023)	Depression, suicidal thoughts, burnout; interventions: psychotherapy suggested	Community‐wide interventions in South Korean healthcare	Workplace relational dynamics and conflicts	Impact of burnout on healthcare quality, workforce stability
Kleinhendler‐Lustig et al. (2023)	Maladaptive perfectionism, internalized standards linked to burnout and depression	Shared emotional strain, need for community mental health resources	Close relationships as protective factors; importance of peer support	Broader implications for healthcare quality, public support during crises
Ofei‐Dodoo et al. (2019)	Burnout affects quality of life and career intentions; wellness practices suggested	Burnout affecting team morale and cohesion in healthcare	Strained relationships with colleagues and patients as risks	Burnout's impact on patient safety and healthcare provider shortage
Lebares et al. (2018)	Mindfulness training as intervention for burnout	Stress in surgical training, importance of supportive environment	Emotional exhaustion impacts patient interactions	Burnout's broader impact on healthcare quality and professionalism
Zisook et al. (2022)	Burnout, depression, risk of suicide and substance abuse; need for mental health interventions	High‐stress culture affecting team dynamics	Emotional exhaustion affects patient and team relationships	Impact on healthcare quality, increased medical errors

## 4 Discussion

This scoping review addressed a critical concern within the healthcare community: the relationship between burnout and depression, and potential links to STB among healthcare professionals.

### 4.1 Relationship Between Burnout, Depression, and Suicide

The relationship between burnout, depression, and suicidal behaviors is evident in many studies, highlighting the intersection of these conditions. Burnout has been frequently associated with a heightened risk of suicidal ideation in the healthcare environment. For instance, Menon et al. (2020) and Williford et al. (2018) identified strong correlations between burnout and suicidal thoughts, with Williford et al. (2018) reporting a six‐fold increase in risk among medical residents experiencing burnout. Yun et al. (2023) also further emphasized this connection, noting burnout was closely associated with thoughts of self‐harm using a multigroup mediation analysis. In short, these findings highlight that burnout not only affects professional performance but also undermines overall mental health.

In addition to burnout's direct association with suicidal behaviors, it often coexists with depression or may predict it. Bai et al. (2022) demonstrated that individuals with higher burnout levels also exhibited more severe depressive symptoms, which in turn contributed to an increased risk of suicide. Samuelsson et al. (1997) provided earlier evidence of this interplay, examining how environmental stressors linked to burnout foster suicidal feelings, though depression was not measured directly. Together, these studies paint a picture of a possible compounding effect: burnout triggering or exacerbating depressive symptoms, and depression elevating the risk of suicidal ideation. This relationship is particularly concerning in high‐stress professions, where burnout and depression are prevalent and often coexist. Authors agree with a previous suggestion that employees with symptoms of burnout also warrant evaluation for depression (Zisook et al. 2022).

### 4.2 Interventions That Address Burnout and Depression to Prevent Suicide

In this scoping review, suggested interventions to address burnout, depression, and suicidal behaviors were provided, yet none of these were tested experimentally, warranting future research. As previously submitted by Rath et al. (2015), Yun et al. (2023) suggest that institutions should encourage work–life balance and reduce job demands that impair quality of life, such as long work hours and excessive or conflicting job demands. Guille and Sen (2024) further endorse workplace modifications and stress the need for health care organizations to create meaningful reductions in workload and to establish and uphold policies that support mental health treatment, family leave, diversity, equity, and inclusion. They also stress the need for researchers to utilize valid assessments of well‐being and depression, determine effective implementation and dissemination strategies for established interventions, and develop and evaluate new targeted interventions. Further, the United States Preventive Services Task Force and a large body of research indicate that cognitive‐behavioral therapy (CBT) or interpersonal therapy should be used as first‐line treatment for individuals with depression (Cuijpers et al. 2016; David et al. 2018; Melnyk 2024; Qaseem et al. 2023; US Preventive Services Task Force et al. 2023).

Yun et al. (2023) notes that assistance in resolving interpersonal conflict as well as a safe place to discuss difficult situations may be beneficial, an idea going back many years as a recommendation for change (Fridner et al. 2009). Kleinhendler‐Lustig et al. (2023) suggest programs to address maladaptive perfectionism to lessen the risk of suicidal ideation.

From the extant literature, the importance of reducing mental health stigma and punitive policies that interfere with receiving treatment and addressing mental health and substance use disorders is well known, as practitioners noted fearing repercussions with their medical licensure (Moutier et al. 2021). The United States Surgeon General further defines interventions that may be helpful to address burnout, such as offering family‐friendly policies like childcare and elder care, paid sick leave and family leave, living wages, and support for educational debt; ensuring workers protection from violence and infection (e.g., supplying adequate personal protective equipment); and lessening job demands by reducing documentation and other administrative burdens (Office of the Surgeon General 2022; Schimmels et al. 2023; Wakefield et al. 2021).

### 4.3 Partial or Incomplete Studies

Only nine articles met the scoping review criteria of addressing relationships between burnout, depression, and suicidal behaviors; however, there were 21 articles that did address burnout, depression, and suicidal behaviors in a manner which did not meet inclusion criteria (Figure 1) yet still provided helpful information. The overall outcomes suggest that burnout may predict depression and depression may predict burnout. Depression was clearly associated with suicidal ideation. In the only study evaluating actual suicide, depression and not burnout predicted both suicide and suicidal ideation. Having children was repeatedly reported as a possible protective factor for suicide. Mixed results were found regarding malpractice litigation as a risk for suicide. Addressing loneliness through social programs may be protective. Proactive offerings of psychological therapy with an opt‐out feature were effective in reducing psychological symptoms in a small pilot of residents. This gives hope that proactive interventions can improve the mental health of the workforce. Results of these 21 studies with key lessons and citations are provided (S2).

### 4.4 OSF Registration Expectations

Our expectations regarding the available research were validated through the review. While many articles are published on the topics of burnout, depression, and STB, only nine met the criteria: all were descriptive in nature, and none included information about self‐harming behaviors, completed suicide, or survivorship. The SESM model can be used as a framework for organizing proposed interventions to guide future research of suicide prevention efforts among healthcare professionals.

### 4.5 Limitations/Future Research

Future research could aim to validate the findings using diagnostic tools that accurately capture the clinical diagnosis of depression, rather than relying primarily on symptom screening tools. Moreover, longitudinal designs and standardized interviews to better understand the temporal relationships among depression symptoms, burnout, and suicidal aspects are needed (Kleinhendler‐Lustig et al. 2023). Further research into phenotypic network analyses could also help elucidate complex relationships in work‐related depression and burnout among healthcare professionals. Given there were no articles studying survivorship from suicidal behaviors for subjects that had both depression and burnout, this is a fertile topic for future research. Most studies included in this review focused on physicians. More studies that include nursing and allied health professionals are needed, as are studies that include all healthcare professionals. Further, papers published after the cutoff for analysis (March 5, 2024) were not included in this review and may provide further insight.

#### 4.5.1 Linking Evidence to Action

- Interventional studies are needed to test strategies addressing burnout, depression, suicidal behaviors, and survivorship of suicide attempts.
- Depression should be considered and evaluated when healthcare workers exhibit symptoms of burnout.
- The Socio‐economic Model of Suicide Prevention (SESM) can be used to categorize suicide prevention measures in healthcare.

## 5 Conclusion

This scoping review highlights a serious gap in knowledge by mapping the sparsely documented existing evidence, identifying the research voids, and providing a confirmation that burnout and depression may intersect with suicidal thoughts and behaviors in healthcare workers. It is not yet fully understood how burnout and depression interact to influence the risk of suicide among healthcare professionals. No evidence was found regarding the relationship between burnout and self‐harming behaviors, suicide, or survivorship when coexisting depression is known. No evidence was found regarding interventions that may enhance survivorship from suicidal ideation among healthcare professionals. The SESM model is applicable to healthcare. Further research is necessary to inform intervention strategies and healthcare policy aimed at mitigating the risk of healthcare professional suicide. The findings of this review serve as a foundation for future research and to test practical interventions designed to address these critical issues in healthcare settings.

## Conflicts of Interest

The authors declare no conflicts of interest.

## Supporting information


Appendix S1.

Appendix S2.

## Data Availability

The data that supports the findings of this study are available in the supplementary material of this article.
