# Supplementary material for: The Relationship Between Depression, Burnout, and Suicide Among Healthcare Professionals: A Scoping Review
Source: Worldviews Evid Based Nurs. 2025 May 13;22(3):e70037. doi: 10.1111/wvn.70037 (PMC12075674; doi:10.1111/wvn.70037)
Supplement: Supplementary file 1 — Appendix S1. [file WVN-22-0-s001.docx]

SUPPLEMENTARY MATERIAL 1

Search Strategy: PubMed

**Concept 1:** Burnout

("Burnout, Professional"[Mesh] OR burnout)

**Concept 2**: Depression

("Depression"[Mesh] OR "depressive disorder" OR "clinical depression" OR "Mental Health"[Mesh] OR Depress*)

**Concept 3:** Suicidal Aspects

("Suicidal Ideation"[Mesh] OR "Suicide, Attempted"[Mesh] OR "Self-Injurious Behavior"[Mesh] OR suicide attempt* OR "self-harm" OR suicide ideation* OR suicidal ideation*)

**Concept 4:** Healthcare workers

(physicians OR nurses OR pharmacists OR "healthcare workers")

**Here is the complete search statement formed by joining the 4 concepts with AND:**

Results: 122

(burnout OR

"burnout syndrome" OR "professional burnout" OR (MH "Depression+") AND (MH "Burnout,

Professional+")) AND (depression OR

"clinical depression" OR "major depression" OR

"depressive disorder" OR (MH "Depression") OR (MH "Depression, Reactive") OR (MH "Dysthymic Disorder")) AND ("suicidal ideation" OR "suicide attempts" OR "self-harm" OR "self-injury" OR (MH "Suicide+") OR (MH "Suicidal Ideation") OR (MH "Suicide Prevention") OR (MH "Suicide, Attempted") OR (MH "Self-Injurious Behavior") OR (MH "Injuries, Self-Inflicted"))

AND  ((MH "Health Personnel+")  OR "healthcare professionals" OR "healthcare workers" OR physicians OR pharmacists OR nurses OR "medical staff"))

Search Strategy: CINAHL

**Concept 1:** Burnout

(burnout OR "burnout syndrome" OR "professional burnout" OR (MH "Burnout, Professional+"))

**Concept 2**: Depression

(depression OR "clinical depression" OR "major depression" OR "depressive disorder" OR (MH "Depression") OR (MH "Depression, Reactive") OR (MH "Dysthymic Disorder"))

**Concept 3:** Suicidal Aspects

("suicidal ideation" OR "suicide attempts" OR "self-harm" OR "self-injury" OR (MH "Suicide+") OR (MH "Suicidal Ideation") OR (MH "Suicide Prevention") OR (MH "Suicide, Attempted") OR (MH "Self-Injurious Behavior") OR (MH "Injuries, Self-Inflicted"))

**Concept 4:** Healthcare workers

((MH "Health Personnel+") OR "healthcare professionals" OR "healthcare workers" OR physicians OR pharmacists OR nurses OR "medical staff")

**Here is the complete search statement formed by joining the 4 concepts with AND:**

Results: 122

(burnout OR

"burnout syndrome" OR "professional burnout" OR (MH "Depression+") AND (MH "Burnout,

Professional+")) AND (depression OR

"clinical depression" OR "major depression" OR

"depressive disorder" OR (MH "Depression") OR (MH "Depression, Reactive") OR (MH "Dysthymic Disorder")) AND ("suicidal ideation" OR "suicide attempts" OR "self-harm" OR "self-injury" OR (MH "Suicide+") OR (MH "Suicidal Ideation") OR (MH "Suicide Prevention") OR (MH "Suicide, Attempted") OR (MH "Self-Injurious Behavior") OR (MH "Injuries, Self-Inflicted"))

AND  ((MH "Health Personnel+")  OR "healthcare professionals" OR "healthcare workers" OR physicians OR pharmacists OR nurses OR "medical staff"))

Search Strategy: PsycInfo

**Concept 1:** Burnout

(MAINSUBJECT.EXACT.EXPLODE("Occupational Stress") OR MAINSUBJECT.EXACT("Burnout") OR "Professional Burnout" OR burnout)

**Concept 2**: Depression

(MAINSUBJECT.EXACT("Dysthymic Disorder") OR MAINSUBJECT.EXACT("Reactive Depression") OR MAINSUBJECT.EXACT("Late Life Depression") OR MAINSUBJECT.EXACT("Recurrent Depression") OR MAINSUBJECT.EXACT("Major Depression") OR MAINSUBJECT.EXACT("Depression (Emotion)") OR MAINSUBJECT.EXACT("Atypical Depression") OR "Depression" OR "depressive disorder" OR "clinical depression")

**Concept 3:** Suicidal Aspects

(MAINSUBJECT.EXACT("Attempted Suicide") OR MAINSUBJECT.EXACT.EXPLODE("Suicide") OR MAINSUBJECT.EXACT("Assisted Suicide") OR MAINSUBJECT.EXACT("Suicide Prevention") OR "Suicidal Ideation" OR "Suicide Attempts" OR "Self-Injurious Behavior" OR "suicide attempts" OR "self-harm")

**Concept 4:** Healthcare workers

(MAINSUBJECT.EXACT.EXPLODE("Nurses") OR MAINSUBJECT.EXACT("Pharmacists") OR MAINSUBJECT.EXACT.EXPLODE("Physicians") OR MAINSUBJECT.EXACT.EXPLODE("Health Personnel") OR physicians OR nurses OR pharmacists OR "healthcare workers")

**Here is the complete search statement formed by joining the 4 concepts with AND:**

Results: 77

((MAINSUBJECT.EXACT.EXPLODE("Occupational Stress") OR MAINSUBJECT.EXACT("Burnout") OR "Professional Burnout" OR burnout) AND (MAINSUBJECT.EXACT("Attempted Suicide") OR MAINSUBJECT.EXACT.EXPLODE("Suicide") OR MAINSUBJECT.EXACT("Assisted Suicide") OR MAINSUBJECT.EXACT("Suicide Prevention") OR "Suicidal Ideation" OR "Suicide Attempts" OR "Self-Injurious Behavior" OR "suicide attempts" OR "self-harm") AND ((MAINSUBJECT.EXACT("Dysthymic Disorder") OR MAINSUBJECT.EXACT("Reactive Depression") OR MAINSUBJECT.EXACT("Late Life Depression") OR MAINSUBJECT.EXACT("Recurrent Depression") OR MAINSUBJECT.EXACT("Major Depression") OR MAINSUBJECT.EXACT("Depression (Emotion)") OR MAINSUBJECT.EXACT("Atypical Depression")) OR ("Depression" OR "depressive disorder" OR "clinical depression")) AND ((MAINSUBJECT.EXACT.EXPLODE("Nurses") OR MAINSUBJECT.EXACT("Pharmacists") OR MAINSUBJECT.EXACT.EXPLODE("Physicians") OR MAINSUBJECT.EXACT.EXPLODE("Health Personnel")) OR (physicians OR nurses OR pharmacists OR "healthcare workers"))) AND la.exact("ENG")
