# Supplementary material for: The Relationship Between Depression, Burnout, and Suicide Among Healthcare Professionals: A Scoping Review
Source: Worldviews Evid Based Nurs. 2025 May 13;22(3):e70037. doi: 10.1111/wvn.70037 (PMC12075674; doi:10.1111/wvn.70037)
Supplement: Supplementary file 2 — Appendix S2. [file WVN-22-0-s002.docx]

**SUPPLEMENTARY MATERIAL 2** Partial knowledge.

| Author | Title | Participants | Findings |
| --- | --- | --- | --- |
| Höller  2022 | Ambivalent heroism? - Psychological burden and suicidal ideation among nurses during the Covid-19 pandemic. | Population: Nurses in Germany  Sample Size: 1311 | Depression was found to be not only associated with suicidal ideation but also a significant predictor of suicidal ideation. Burnout was not found to be a significant predictor of suicidal ideation. Nurses in the sample population with high resilience suffered significantly less from burnout and depression in comparison to nurses with medium to low resilience. |
| Ho  2023 | Cross-sectional survey of sleep, fatigue and mental health in veterinary anesthesia personnel. | Population: Mixed veterinary workers around the globe  Sample Size: 393 | Suicidal ideation was associated with major depression, as well as burnout. Burnout was found to have an association with major depression. |
| Korkeila  2003 | Burnout and self-perceived health among Finnish psychiatrists and child psychiatrists: A national survey. | Population: Physicians in Finland  Sample Size: 3133 | Positive correlation between burnout and depression found. Depression (self-diagnosed) was found to be appreciably associated with overall score on the Maslach Burnout Inventory. |
| Zisook  2024 | The association of psychotherapy with burnout, depression, and measures of well-being in residents and fellows: A pilot study. | Population: Physicians in the United States  Sample Size: 30 | Showed the Healer Education Assessment & Referral Program's resident therapy program resulted in notable decreases of depression severity and burnout as time passed. No notable effects to suicidal ideation were observed in the duration from baseline to the end of the 12-week treatment phase. |
| Kwok  2021 | Depression, stress, and perceived medical errors in Singapore psychiatry residents | Population: Physicians in Singapore  Sample Size: 47 | Showed those who scored higher in depersonalization and emotional exhaustion (used to indicate burnout) had higher PHQ-9 scores. Results did not indicate if the higher PHQ-9 were positive screenings for depression in those with burnout. 33% reported feeling burnout, 12% depression, and 10.6% positive for suicidal ideation. |
| Rath  2015 | Burnout and associated factors among members of the Society of Gynecologic Oncology. | Population: Mixed healthcare workers in the United States  Sample Size: 436 | Showed those with positive depression screening had higher odds of burnout. Recommends interventions to address burnout by focusing on quality of life, treatment of depression, and/or alcohol abuse. Although almost half the respondents noted reluctance to seek help. |
| Hopcraft  2023 | Factors associated with suicidal ideation and suicide attempts by Australian dental practitioners. | Population: Mixed healthcare workers in Australia  Sample Size: 1474 | Suicide was correlated with a higher rate of depression but not associated with burnout. |
| Sterud  2008 | Suicidal ideation and suicide attempts in a nationwide sample of operational Norwegian ambulance personnel | Population: Mixed healthcare workers in Norway  Sample Size: 1286 | Depression symptoms significantly associated with serious suicidal ideation (SI); job related emotional exhaustion (measurement of burnout) associated with higher levels of serious SI; depression symptoms also significant for positive association for serious SI |
| Faivre  2018 | Burnout syndrome in orthopaedic and trauma surgery residents in France: A nationwide survey | Population: Physicians in France  Sample Size: 107 | Found burnout to be independently and significantly associated with depression. |
| Kramper  2023 | Highly stressful events and posttraumatic stress disorder symptoms among veterinary professionals: Prevalence and associations with mental health and job-related outcomes | Population: Mixed veterinary workers in the United States  Sample Size: 359 | Showed the more stressful events a veterinarian had, the more likely they would have burnout, depression or suicidal ideation. Recommend veterinarians be evaluated for mental health. |
| Ménard  2023 | One‐year follow‐up of hospital nurses' work experiences during the COVID‐19 pandemic: A qualitative study | Population: Nurses in Canada  Sample Size: 19 | Assessed factors associated with loneliness in medical trainees. Burnout, depression, and suicidal ideation were all associated with loneliness. Authors advised interventions that address loneliness might be helpful in reducing incidence of burnout, depression and suicidal ideation. |
| Al-Humadi  2021 | Depression, suicidal thoughts, and burnout among physicians during the COVID-19 pandemic: A survey-based cross-sectional study | Population: Physicians in the United States  Sample Size: 225 | Authors reported an association between burnout, depression, and suicidal ideation as it relates to prior known anxiety/depression. They recommend mental health screening for physicians given the prevalence of mental health symptoms present. |
| McCloskey  2022 | Ohio physicians' retrospective pre-post COVID-19 pandemic reports of burnout and well-being. | Population: Physicians in the United States  Sample Size: 7164 | Demonstrated burnout, depression and suicidal ideation were all strongly associated with malpractice litigation. Authors stated more research needed to test interventions to protect physicians experiencing malpractice litigation. |
| Grover  2018 | Psychological problems and burnout among medical professionals of a tertiary care hospital of North India: A cross-sectional study. | Population: Physicians in North India  Sample Size: 1721 | Mental health issues were higher among residents and faculty and associated with verbal/physical abuse and whether senior faculty showed empathy to the residents or patients. Longer work hours were associated with higher burnout, depression and stress. Residents had higher work hours than faculty.  60% were concerned about seeking treatment because of threats to their medical license. Authors suggest routine screening for depression, reducing stigma against seeking help, and reducing work hours. |
| Fridner  2009 | Survey on recent suicidal ideation among female university hospital physicians in Sweden and Italy (the HOUPE study): Cross-sectional associations with work stressors. | Population: Physicians in Italy and Sweden  Sample Size: 511 | Demonstrated a high incidence of physicians who had previously sought help for either burnout or depression (12-20%), had suicidal ideation within the last 12 months (14%) and had ever had suicidal ideation (21-33%). The Odds ratio for suicidal thoughts was higher if the physician had sought help for burnout or depression. Authors contributed work conditions to differences in results between countries when analyzed separately, suggesting interventions need to be culturally relevant to be protective. Having time set aside to discuss difficult situations at work was universally protective. |
| Bismark  2022 | Thoughts of suicide or self-harm among Australian healthcare workers during the COVID-19 pandemic. | Population: Mixed healthcare workers in Australia  Sample Size: 7795 | Found approximately 10% of respondents had suicidal ideation. More common in workers who reported burnout and depression. Nearly all had some evidence of depression (97%), with 49% having moderate to severe symptoms. More than half of those with suicidal ideation had not sought help for their condition. Authors suggest more attention needs to be focused on encouraging help-seeking behaviors among healthcare workers. |
| Shanafelt  2021 | Suicidal ideation and attitudes regarding help seeking in US physicians relative to the US working population. | Population: Physicians in the United States  Sample Size: 5197 | 6.5% of physicians had suicidal ideation within the last year. Those with suicidal ideation were less likely to seek mental health treatment than others. Emotional exhaustion aspect of burnout and depression were associated with suicidal ideation. There was no relationship between recent malpractice and suicidal ideation. Authors caution stigma, in particular threats against career and license, should be addressed to overcome the barrier to help-seeking behaviors. |
| Fresán  2019 | Professional adversities and protective factors associated with suicidal ideation in Mexican psychiatrists. | Population: Physicians in Mexico  Sample Size: 233 | Showed burnout and depression independently were the strongest predictors of suicidal ideation. Suicidal ideation was reported in 7.6% of respondents. Social support, being married, having children, and other physicians in the family were protective factors. |
| Shanafelt  2011 | Special report: Suicidal ideation among American surgeons. | Population: Physicians in the United States  Sample Size: 7501 | This survey showed 6% of respondents had suicidal ideation. Burnout and depression were independently significant predictors of suicidal ideation. The incidence of depression was like that of the general population, but suicidal ideation was higher. Authors posited burnout may be an important factor driving suicidal ideation. A minority of those with mental health symptoms had sought medical treatment. A third of physicians reported fear in seeking treatment, because of threats to their medical license. |
| Lazarescu  2018 | Prevalence of burnout, depression and job satisfaction among French senior and resident radiation oncologists. | Population: Physicians in France  Sample Size: 242 | Depression and suicidal ideation were higher in those that exhibited symptoms of burnout. The burnout incidence was 69%. The odds ratio of suicidal ideation was higher with the emotional exhaustion component of burnout. Having children was seen as a protective factor against burnout. Burnout was higher among residents than senior faculty. |
| Faivre  2019 | Are French orthopedic and trauma surgeons affected by burnout? Results of a nationwide survey. | Population: Physicians in France  Sample Size: 441 | Of the respondents, 8% had suicidal ideation and 39% had burnout. Depressive symptoms were predictive of burnout. |

Holler, I. and Forkmann, T. (2022). Ambivalent heroism? – Psychological burden and suicidal ideation among nurses during the Covid‐19 pandemic. *Nursing Open, 9*(1). 785-800. 10.1002/nop2.1130

Ho, N., Santoro, F., Palacios Jimenez, C., and Pelligand, L. (2023). Cross-sectional survey of sleep, fatigue and mental health in veterinary anesthesia personnel. *Veterinary Anaesthesia and Analgesia, 50*(4). 315-324. 10.1016/j.vaa.2023.03.003

Korkeila, J., Töyry, S., Kumpulainen, K., Toivola, J., Räsänen, K., and Kalimo, R. (2003). Burnout and self-perceived health among Finnish psychiatrists and child psychiatrists: A national survey. *Scandinavian Journal of Public Health, 31*(2). 85-91. 10.1080/14034940210133880

Zisook, S., Doran, N., Downs, N., Shapiro, D., Haddad, A., Lee, D., Newton, I., Kawaski, J., Nestsiarovich, A. and Davidson, J. (2024). The association of psychotherapy with burnout, depression, and measures of well-being in residents and fellows: A pilot study. *Academic Medicine.* 10.1097/ACM.0000000000005750

Kwok, C. (2021). Depression, stress, and perceived medical errors in Singapore psychiatry residents. *Academic Psychiatry, 45*(2). 169-73. 10.1007/s40596-020-01376-w

Rath, K., Huffman, L., Phillips, G., Carpenter, K., and Fowler, J. (2015). Burnout and associated factors among members of the Society of Gynecologic Oncology. *American Journal of Obstetrics and Gynecology, 213*(6). 824.e1-824.e9. 10.1016/j.ajog.2015.07.036

Hopcraft, M., Stormon, N., McGrath, R., and Parker, G. (2015). Factors associated with suicidal ideation and suicide attempts by Australian dental practitioners. *Community Dentistry and Oral Epidemiology, 51*(6). 1159-1168. 10.1111/cdoe.12849

Sterud, T., Hem, E., Lau, B., and Ekeberg, Ø. (2008). Suicidal ideation and suicide attempts in a nationwide sample of operational Norwegian ambulance personnel. *Journal of Occupational Health, 50*(5). 406-414. 10.1539/joh.l8025

Faivre, G., Kielwasser, H., Bourgeois, M., Panouilleres, M., Loisel, F., Obert, L. (2018). Burnout syndrome in orthopaedic and trauma surgery residents in France: A nationwide survey. *Orthopaedics & Traumatology, Surgery & Research, 104*(8). 1291-1295. 10.1016/j.otsr.2018.08.016

Kramper, S., Crosby, E., Waitz-Kudla, S., Weathers, F., Witte, T., and Kendall-Tackett, K. (2023). Highly stressful events and posttraumatic stress disorder symptoms among veterinary professionals: Prevalence and associations with mental health and job-related outcomes. *Psychological Trauma, 15*(S2). S275-S285. 10.1037/tra0001432

Ménard, A., Soucie, K., Ralph, J., Chang, Y., Morassutti, O., Foulon, A., Jones, M., Desjardins, L., and Freeman, L. (2023). One‐year follow‐up of hospital nurses' work experiences during the COVID‐19 pandemic: A qualitative study. *Journal of Advanced Nursing, 79*(7). 2502- 2513. 10.1111/jan.15599

Al-Humadi, S., Bronson, B., Muhlrad, S., Paulus, M., Hong, H., and Cáceda, R. (2021). Depression, suicidal thoughts, and burnout among physicians during the COVID-19 pandemic: A survey-based cross-sectional study. *Academic Psychiatry, 45*(5). 557-565. 10.1007/s40596-021-01490-3

McCloskey, R., Hammond, G., Gallant, K., Santucci, R., Koralewski, J., and Kochinski, M. (2022). Ohio physicians' retrospective pre-post COVID-19 pandemic reports of burnout and well-being. *Journal of Medical Regulation, 108*(3). 8-17. 10.30770/2572-1852-108.3.8

Grover, S., Sahoo, S., Bhalla, A., and Avasthi, A. (2018). Psychological problems and burnout among medical professionals of a tertiary care hospital of North India: A cross-sectional study. *Indian Journal of Psychiatry, 60*(2). 175-188. 10.4103/psychiatry.IndianJPsychiatry_254_17

Fridner, A., Belkic, K., Marini, M., Minucci, D., Pavan, L., and Schenck-Gustafsson, K. (2009). Survey on recent suicidal ideation among female university hospital physicians in Sweden and Italy (the HOUPE study): cross-sectional associations with work stressors. *Gender Medicine, 6*(1). 314-328. 10.1016/j.genm.2009.04.006

Bismark, M., Scurrah, K., Pascoe, A., Willis, K., Jain, R., and Smallwood, N. (2022). Thoughts of suicide or self-harm among Australian healthcare workers during the COVID-19 pandemic. *Australian and New Zealand Journal of Psychiatry, 56*(12). 1555-1565. 10.1177/00048674221075540

Shanafelt, T., Dyrbye, L., West, C., Sinsky, C., Tutty, M., Carlasare, L., Wang, H., and Trockel, M. (2021). Suicidal ideation and attitudes regarding help seeking in US physicians relative to the US working population. *Mayo Clinic Proceedings, 96*(8). 2067-2080. 10.1016/j.mayocp.2021.01.033

Fresán, A., Yoldi-Negrete, M., Robles-García, R., Tovilla-Zárate, C., and Suárez-Mendoza, A. (2019). Professional adversities and protective factors associated with suicidal ideation in Mexican psychiatrists. *Archives of Medical Research, 50*(8). 484-489. 10.1016/j.arcmed.2019.11.010

Shanafelt, T., Balch, C., Dyrbye, L., Bechamps, G., Russell, T., Satele, D., Rummans, T., Swartz, K., Novotny, P., Sloan, J., and Oreskovich, M. (2011). Special report: Suicidal ideation among American surgeons. *Archives of Surgery, 146*(1). 54-62. 10.1001/archsurg.2010.292

Lazarescu, I., Dubray, B., Joulakian, M.B., Blanchard, P., Chauvet, B., Mahé, M.-A., Mornex, F., Rocher, F., and Thureau, S. (2018). Prevalence of burnout, depression and job satisfaction among French senior and resident radiation oncologists. *Cancer*  *Radiothérapie, 22*(8). 784-789. 10.1016/j.canrad.2018.02.005

Riley, R., Spiers, J., Chew-Graham, C., Taylor, A., Thornton, G., and Buszewicz, M. (2019). Are French orthopedic and trauma surgeons affected by burnout? Results of a nationwide survey. *BMJ Open, 8*(5). e018620-e018620. 10.1136/bmjopen-2017-018620
